# Gliadin, through the Activation of Innate Immunity, Triggers lncRNA NEAT1 Expression in Celiac Disease Duodenal Mucosa

**DOI:** 10.3390/ijms22031289

**Published:** 2021-01-28

**Authors:** Elisa Gnodi, Clara Mancuso, Luca Elli, Elisa Ballarini, Raffaella Meneveri, Jean François Beaulieu, Donatella Barisani

**Affiliations:** 1 School of Medicine and Surgery, University of Milano-Bicocca, 20900 Monza, Italy; e.gnodi@campus.unimib.it (E.G.); clara.mancuso@unimib.it (C.M.); elisa.ballarini@unimib.it (E.B.); raffaella.meneveri@unimib.it (R.M.); 2 Centre for the Prevention and Diagnosis of Celiac Disease, Gastroenterology and Endoscopy Unit, Fondazione IRCCS Ca’ Granda Ospedale Maggiore Policlinico, 20122 Milan, Italy; luca.elli@policlinico.mi.it; 3 Laboratory of Intestinal Physiopathology, Faculty of Medicine and Health Sciences, Université de Sherbrooke and Research Center of the Centre Hospitalier Universitaire de Sherbrooke, Sherbrooke, QC J1H 5N4, Canada; jean-francois.beaulieu@usherbrooke.ca

**Keywords:** celiac disease, long non-coding RNAs, NEAT1, TUG1, innate immunity

## Abstract

Celiac disease (CD) is an autoimmune enteropathy arising in genetically predisposed subjects exposed to gluten, which activates both innate and adaptive immunity. Although the pathogenesis is common to all patients, the clinical spectrum is quite variable, and differences could be explained by gene expression variations. Among the factors able to affect gene expression, there are lncRNAs. We evaluated the expression profile of 87 lncRNAs in CD vs. healthy control (HC) intestinal biopsies by RT-qPCR array. Nuclear enriched abundant transcript 1 (NEAT1) and taurine upregulated gene 1 (TUG1) were detected as downregulated in CD patients at diagnosis, but their expression increased in biopsies of patients on a gluten-free diet (GFD) exposed to gluten. The increase in NEAT1 expression after gluten exposure was mediated by IL-15 and STAT3 activation and binding to the NEAT1 promoter, as demonstrated by gel shift assay. NEAT1 is localized in the nucleus and can regulate gene expression by sequestering transcription factors, and it has been implicated in immune regulation and control of cell proliferation. The demonstration of its regulation by gluten thus also supports the role of lncRNAs in CD and prompts further research on these RNAs as gene expression regulators.

## 1. Introduction

Celiac disease is a multifactorial autoimmune disorder present in 1% of the worldwide population, with regional differences due to different genetic backgrounds in diverse ethnic groups as well as environmental factors. The genetic background necessary for CD development is represented by specific antigen presentation molecules (MHC-II), namely HLA-DQ2/DQ8 haplotypes, whereas the triggering factor is gluten, commonly found in several cereals such as wheat, barley and rye. When gluten peptides arrive in the duodenum of a predisposed subject after a partial digestion in the stomach, they pass through the intestinal barrier and cause IL-15 production that, in turn, activates innate immunity in the epithelium and adaptive immunity in the lamina propria, with the production of various cytokines, such as IFNγ [1], leading to mucosal destruction with crypt hyperplasia and villous atrophy. A life-long gluten-free diet is the only current therapy for CD, which can restore a normal intestinal mucosa in most patients [2].

Although research has clarified several mechanisms involved in CD pathogenesis, there are still questions regarding various aspects of the disorder, including which factors determine the age of development, the severity of the lesion or the response to GFD. The regulation of gene expression can have an important role in determining clinical features, the pivotal role of non-coding RNAs in this process is now understood. In recent years, attention has been mainly focused on microRNAs, which have been found to be deregulated in CD, with different microRNA panels correlated to the age of onset or with different stages of CD [3,4]. On the other hand, long non-coding RNAs (lncRNAs) in CD have not been extensively studied yet; these molecules are defined as non-coding transcripts of more than 200 base pairs, localized all over the genome, with various mechanisms of action that mainly result in gene expression regulation, such as chromatin remodeling (i.e., XIST during X-chromosome inactivation), transcription factor (TF) sequestration, direct sequence interaction or microRNA sponging [5,6]. LncRNAs have mainly been studied in cancer, since many of them seem to be deregulated in tumor tissues and linked with cell proliferation, tissue invasion and inflammation [7,8], but also in autoimmune diseases like rheumatoid arthritis or multiple sclerosis [9,10]. In the gut, H19 could regulate intestinal permeability in inflammatory bowel diseases (IBDs) [11,12], whereas lnc13 and HGC14 have been linked with CD [13,14], but their proper function in the disease has not been fully characterized to date.

To further understand the role of lncRNAs in CD pathogenesis and/or in determining the phenotype, we evaluated the expression of a series of lncRNAs in RNA extracted from duodenal biopsies of subjects with active CD and compared it with that of those derived from healthy subjects. Among the differentially expressed genes, we focused on NEAT1 and TUG1, due to their potential role in known pathogenic mechanisms that lead to CD development. In fact, both NEAT1 and TUG1 have also been linked to autoimmune diseases, such as lupus [15,16], multiple sclerosis [17] and rheumatoid arthritis [18]. However, these lncRNAs are known to have different mechanisms of action: NEAT1 forms nuclear bodies called paraspeckles, that can sequester TFs from the promoter region of target genes and regulate their transcription (such as the TF SFPQ from the IL-8 promoter) [19,20], whereas TUG1 mainly acts as a microRNA sponge in the cytosol, interacting with many signaling pathways (i.e., IL-1β, IL-6, IL-10, TNFα) [21,22].

The results reported here demonstrate that the expression of both these lncRNAs is regulated by gluten exposure, but only in CD mucosa, and that this regulation occurs through the production of IL-15 and STAT3 activation in the case of NEAT1.

## 2. Results

### 2.1. Long Non-Coding RNA Expression Varies in CD Mucosa

**Figure 1:**
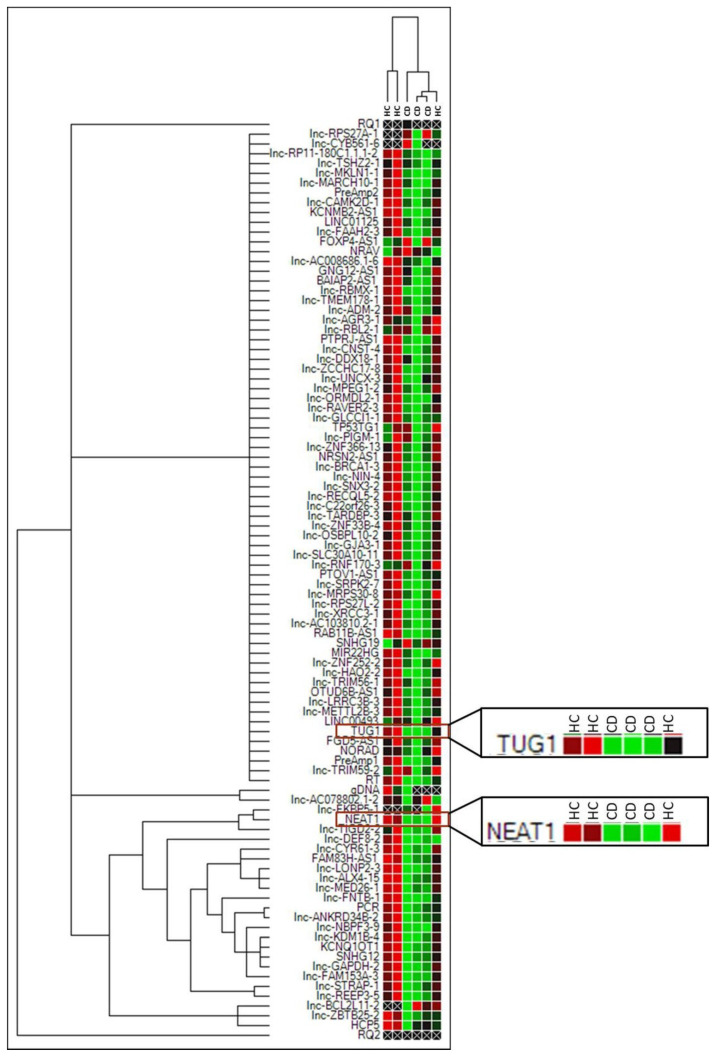
Cluster analysis of the 87 lncRNA array in three Marsh 3C celiac patients (CD) and three healthy controls (HCs). Green = lower expression. Red = higher expression. PreAmp1, PreAmp2, gDNA, PCR, RQ1, RQ2, RT are internal reaction controls.

An initial assessment of lncRNA expression in CD was obtained by performing an array that included 87 lncRNA transcripts on RNA extracted from duodenal biopsies of three newly diagnosed adult Marsh 3C patients and three healthy controls (HCs). Cluster analysis showed a massive downregulation trend in most of the lncRNAs in CD compared to HC samples (Figure 1), whereas an upregulation was observed only for three lncRNAs, namely, FOXP4-AS1, NRAV and SNHG19.

**Figure 2:**
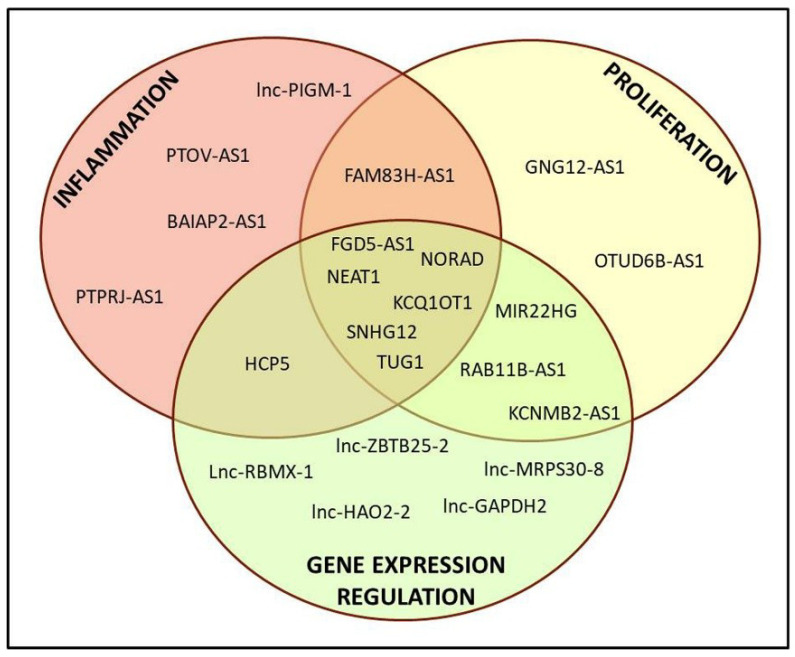
Representative scheme of the derived classification of the 22 lncRNAs with known function.

Among the downregulated lncRNAs, a clear function could be attributed only to a portion of them, i.e., 22 out of 73. A classical Gene Ontology approach is not feasible for lncRNAs, as has also recently been reported [23]; data obtained from various databases allowed us to subdivide them into different categories, namely inflammation, cell proliferation/cancer and gene expression regulation. As shown in Figure 2, various lncRNAs belonged to more than one category, underlying the difficulty in dissecting their proper role in the cell. 

**Figure 3:**
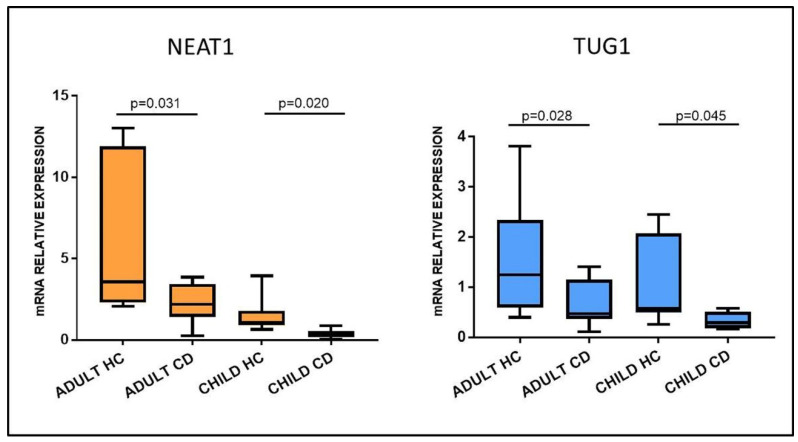
NEAT1 and TUG1 mRNA expression in duodenal biopsies of adult and pediatric Marsh 3C celiac patients (CD) compared to matched healthy controls (HCs). Adult HCs *n* = 7; adult CD *n* = 10; child HCs *n* = 10; child CD *n* = 6. Data are expressed as box plots indicating 25–75th percentile and median value, with whiskers representing 5th and 95th percentile.

Among the many lncRNAs investigated, we chose to focus on NEAT1 and TUG1 because of their reported involvement in autoimmune disorders, for their interactions with immune-related molecules and their role in gene expression regulation. To confirm the array data, NEAT1 and TUG1 expression was investigated in larger cohorts of adult and pediatric patients with RT-qPCR. The results highlighted a significant downregulation in both adult and pediatric Marsh 3C CD patients compared to their respective HC group (Figure 3). In fact, the mean expression of NEAT1 and TUG1 in adult HCs was 5.87 ± 4.64 and 1.64 ± 1.19, respectively, and 2.27 ± 1.24 and 0.65 ± 0.44 in CD patients (*p* = 0.031 and *p* = 0.028, respectively). Pediatric patients had a similar trend, with NEAT1 and TUG1 levels being 1.46 ±0.96 and 1.13 ±0.86 in HCs and 0.39 ± 0.29 and 0.34 ± 0.16 in CD, *p* = 0.020 and *p* = 0.045, respectively.

### 2.2. Gluten Exposure Alters NEAT1 and TUG1 Expression 

**Figure 4:**
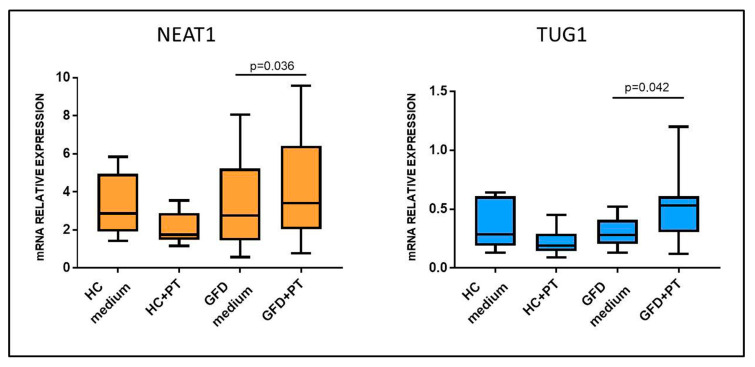
NEAT1 and TUG1 mRNA expression in biopsies of HCs and celiac patients on a gluten-free diet (GFD) after incubation with medium with or without PT-gliadin 1 mg/mL. HCs *n* = 6; GFD *n* = 6. Data are expressed as box plots indicating 25–75th percentile and median value, with whiskers representing 5th and 95th percentile. Data were compared by paired *t*-test.

To assess whether NEAT1 and TUG1 changes in expression could be induced by gluten exposure, pepsin–trypsin-digested gliadin (PT-gliadin), the immunogenic fraction of gluten, was added to incubation medium and used to stimulate biopsies from celiac patients on a gluten-free diet, therefore with a restored normal mucosa (Marsh 0), and HCs. Opposite from what was expected, exposure to PT-gliadin upregulated both NEAT1 and TUG1 expression with respect to simple medium in patients on a GFD (4.27 ± 2.78 vs. 3.30 ± 2.44, for NEAT1, *p* = 0.036; 0.51 ± 0.30 vs. 0.30 ± 0.13 for TUG1, *p* = 0.042), whereas this did not happen in HCs (NEAT1: HC medium 3.29 ± 1.66; HC+PT 2.07 ± 0.87; TUG1: HC medium 0.36 ± 0.21; HC+PT 0.22 ± 0.12), showing that these lncRNAs are induced by gluten exposure only in CD patients (Figure 4). 

### 2.3. NEAT1 and TUG1 Are Preferentially Expressed in the Epithelium

**Figure 5:**
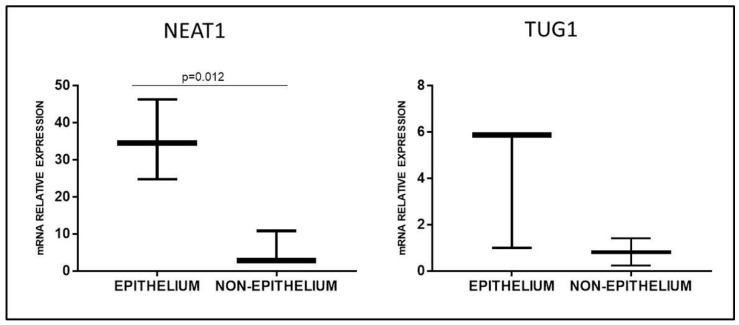
NEAT1 and TUG1 mRNA expression in epithelial or non-epithelial fraction of healthy duodenal mucosa obtained by laser microdissection (*n* = 3). Data are expressed as box plots indicating 25–75th percentile and median value, with whiskers representing 5th and 95th percentile.

Since Marsh 3C patients and PT-stimulated GFD biopsies presented an opposite trend in NEAT1 and TUG1 expression, we hypothesized a different localization of these two lncRNAs in the duodenal tissue. Thus, we performed a laser microdissection on HC biopsies to separate the epithelial from the non-epithelial component; RT-qPCR data showed a preferential expression of NEAT1 and TUG1 in the epithelium (Figure 5), a difference that reached significance in the case of NEAT1 (*p* = 0.012) but not TUG1 (*p* = 0.2). Since most of the epithelium is usually destroyed in mucosa with Marsh 3C lesions, this could explain the opposite trend observed in biopsies obtained at diagnosis or after a gluten-free diet, since in the latter, the epithelium is restored.

### 2.4. NEAT1 and TUG1 Expression Is Triggered by Cytokines

The increase in NEAT1 and TUG1 expression was observed only in CD biopsies after ex vivo stimulation, suggesting the presence of specific factors produced in patients’ mucosa after exposure to PT-gliadin, with interleukins of both innate and adaptive immunity being possible candidates. We thus evaluated their expression in the stimulated biopsies and detected a significant increase in IL-15 production (2.65 ± 1.61 vs. 1.45 ± 0.7; *p* = 0.049) and an increasing trend in IFNγ and IL-8 only in CD samples (data not shown).

**Figure 6:**
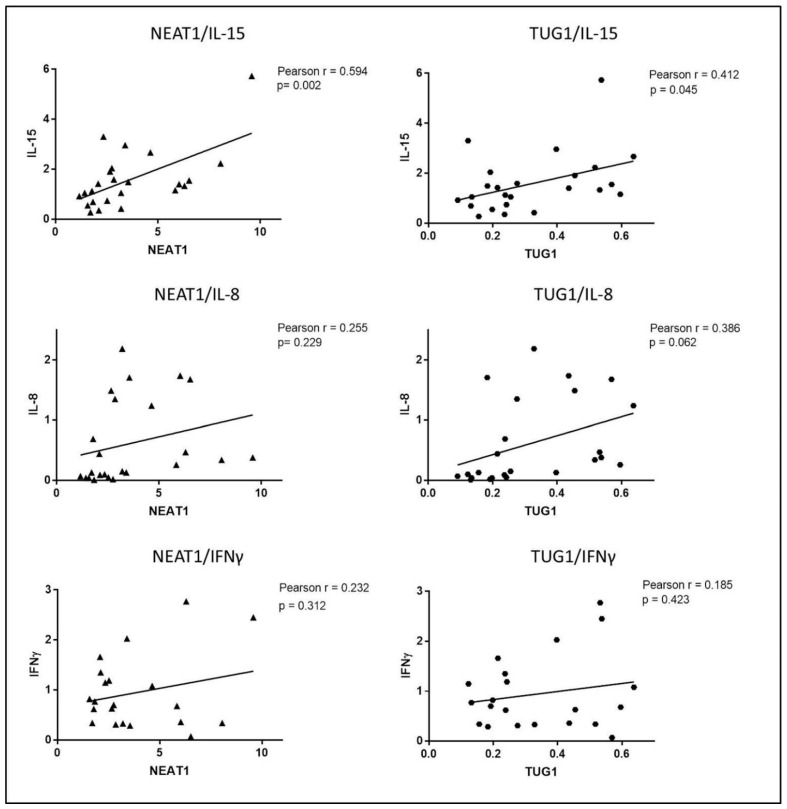
Pearson correlation analysis of mRNA expression levels of NEAT1 and TUG1 with IL-15, IL-8 and IFNγ in biopsies undergoing ex vivo experiments (HCs and GFD patients).

An initial analysis of the possible role of cytokines in regulating NEAT1 and TUG1 expression was performed by Pearson correlation analysis which highlighted a significant positive correlation between NEAT1 and IL-15 (*r* = 0.594, *p* = 0.002), as well as TUG1 and IL-15 (*r* = 0.412, *p* = 0.045) (Figure 6). 

Interestingly, a positive correlation that almost reached significance was also detected between TUG1 and IL-8, whereas neither NEAT1 nor TUG1 expression correlated with IFNγ mRNA levels. Thus, overall, the correlations seem to indicate that NEAT1 and TUG1 expression is linked to the activation of innate immunity rather than adaptive immunity. We also detected a strong positive correlation between NEAT1 and TUG1 (Pearson *r* = 0.819, *p* < 0.0001). 

**Figure 7:**
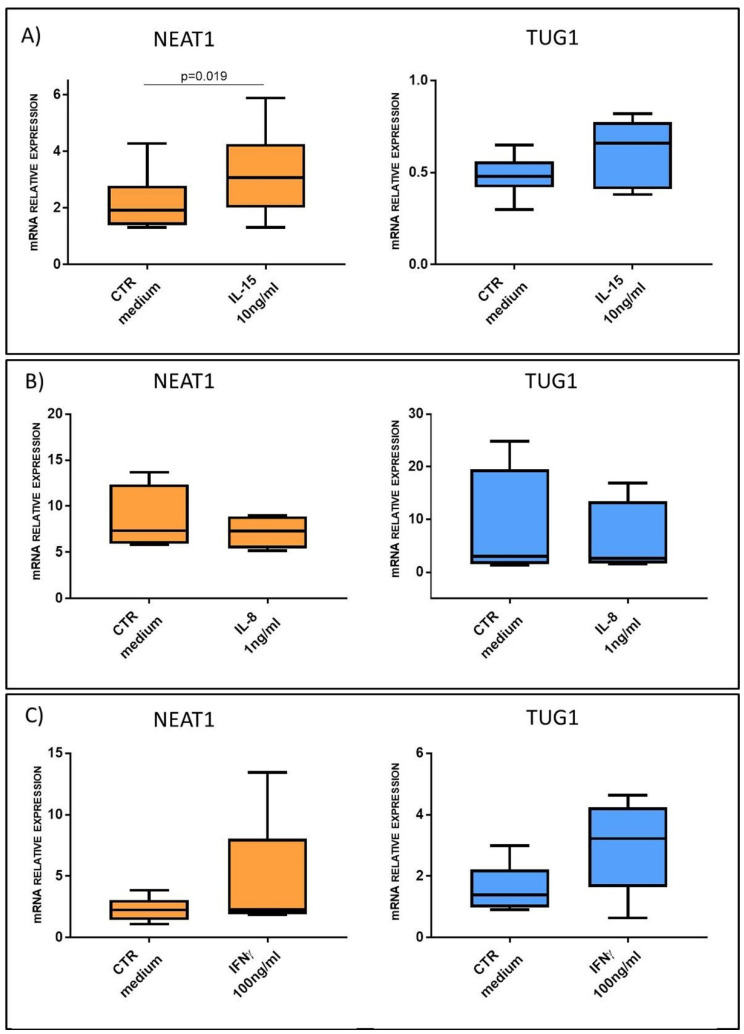
NEAT1 and TUG1 mRNA expression level in HC biopsies after stimulation with or without: (**A**) IL-15 10 ng/mL (*n* = 6); (**B**) IL-8 1 ng/mL (*n* = 4); (**C**) IFNγ 100 ng/mL (*n* = 5). Data are expressed as box plots indicating 25–75th percentile and median value, with whiskers representing 5th and 95th percentile. Data were analyzed by paired *t*-test.

These correlations suggested a possible role of cytokines in the regulation of NEAT1 and TUG1 expression; to further support this hypothesis, we performed an in silico analysis of NEAT1 and TUG1 promoters to identify putative TF binding sites. Different programs (MatInspector, PROMO3, Jaspar) identified several binding sites for the STAT family TFs in both promoters; since STAT3 is downstream of IL-15 (canonical) and IL-8 (non-canonical) signaling pathways [24,25], whereas STAT1 is part of the IFNγ one [26], these cytokines were used to stimulate HC biopsies to evaluate their ability to regulate NEAT1 and TUG1 expression ex vivo. Such stimulations confirmed a link with innate immunity activation; in particular, IL-15 stimulation was able to induce a significant upregulation of NEAT1 (3.22 ± 1.59 vs. 2.19 ± 1.08 in IL-15-treated samples and controls, respectively, *p* = 0.019), whereas IL-8 did not. As regards TUG1, we could not detect a significant increase in its mRNA level neither after IL-15 or IL-8 stimulation, and we could not observe an interaction with IFNγ-mediated adaptive immunity, since treatment with this cytokine did not significantly change either of the two lncRNA levels (Figure 7).

### 2.5. IL-15 Causes the Binding of STAT3 to NEAT1 Promoter in HIEC-6 Cell Model

**Figure 8:**
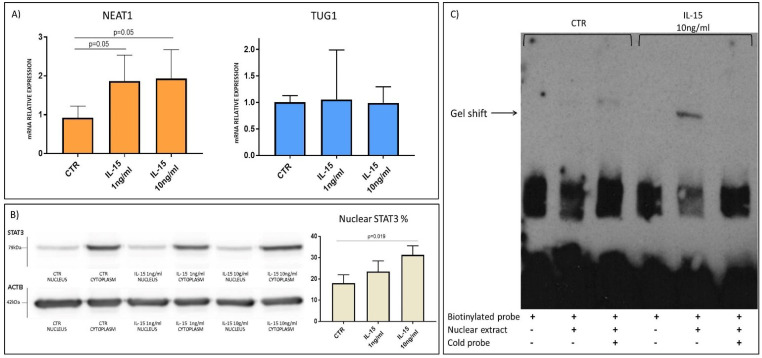
(**A**) NEAT1 and TUG1 mRNA expression level in HIEC-6 cells with or without stimulation with IL-15 (*n* = 3). Data are shown as mean value and standard deviation. (**B**) Translocation of STAT3 in the nucleus after IL-15 stimulation. Each sample was normalized on ACTB. Western blot image representative of three independent experiments. (**C**) DNA retardation assay of nuclear protein extracts with biotinylated oligos of NEAT1 promoter region carrying STAT3 putative binding site. Gel shift band is present after IL-15 stimulation and disappears with excess of cold probe. Assay image representative of three independent experiments.

HIEC-6 intestinal epithelial crypt-like cells were used as an in vitro cell model to better dissect the link between NEAT1 and innate immunity cytokines. Being cells of primary origin [27], they were considered more reliable for lncRNA studies, since other intestinal cell lines are derived from cancers and may have an abnormal expression of NEAT1. Firstly, RT-qPCR confirmed a significant NEAT1 upregulation consequent to IL-15 stimulation, similarly to the ex vivo experiments (Figure 8A). Even in this in vitro model, IL-15 did not induce TUG1 expression. STAT3 translocation into the nucleus in response to hrIL-15 or hrIL-8 stimulation was investigated through western blot on cytosolic and nuclear protein extracts. Stimulation with hrIL-15 induced STAT3 translocation in this cell line (Figure 8B), whereas hrIL-8 did not (Figure S1).

Finally, a DNA retardation assay was performed with HIEC-6 nuclear extracts and biotinylated probes designed on the NEAT1 promoter region carrying the putative binding site for STAT3 (Figure S2). The obtained data confirmed TF binding to the STAT3 binding site caused by IL-15 stimulation, whereas no shift was detected in either the unstimulated sample or with an excess of cold competitor (unbiotinylated oligos) (Figure 8C).

## 3. Discussion

Celiac disease has a prevalence of 1–2% in the Caucasian population, and a lot of effort has been spent in the last decade to further characterize the genetic components necessary for the development of the disease. The first and better-studied genetic locus, HLA class II, accounts for about 40% of the genetic predisposition; various genome-wide association studies (GWAS) performed on different populations have allowed the identification of several additional loci, mostly involved in the regulation of the immune response either at the intestinal or thymic level. Moreover, some of the identified loci were common to other autoimmune disorders, such as IBDs or type I diabetes [2,28]. The identification of these loci and candidate genes did not completely clarify the CD pathogenesis, since the regulation in their expression represents a pivotal process. In fact, a paper that wanted to evaluate the expression of some of the GWAS-identified genes in the intestine of CD patients revealed that their expression was regulated by three single nucleotide polymorphisms (SNPs), with one of these SNPs being localized on a lincRNA, which in turn showed an increased expression in the mucosa in CD patients [29].

LncRNAs can regulate the expression of other genes acting either in cis or in trans, interacting with DNA or with proteins such as transcription factors, but their regulatory function can also occur at a post-transcriptional level, for example, acting as sponges for miRNAs [30]. Although it has been estimated that there are more than 170,000 lncRNAs in humans according to NONCODE v6.0 or 17,000 according to Gencode version 36, only the function of a few of them has been identified [31,32]. It is clear, however, that lncRNAs are involved in several processes that are important in the pathogenesis of CD, such as cell proliferation, apoptosis and inflammatory/immune response [7,33,34,35]. Moreover, an altered expression of various lncRNAs has been detected in other autoimmune disorders, such as IBDs or multiple sclerosis, diseases that share predisposing loci with CD [12,17,36].

An lncRNA associated with susceptibility to CD was identified by Castellanos-Rubio in 2016; lnc13 was able to regulate the expression of different genes involved in inflammation through binding to hnRNPD, a nuclear riboprotein. In addition, lnc13 expression was reduced in the intestinal mucosa of CD patients, allowing an increase in the expression of inflammatory genes [13]. Another lncRNA that was found to be downregulated in the mucosa of CD patients was HCG14, a molecule that was able to regulate the expression of NOD1, a member of the NOD-like receptor family, i.e., a protein in charge of the recognition of bacterial peptidoglycans [14].

In this paper, we analyzed various lncRNAs through an RT-qPCR array, comparing their expression in the duodenal mucosa of Marsh 3C patients vs. controls. Similarly to what happens in other autoimmune disorders, such as multiple sclerosis or rheumatoid arthritis, our results showed an altered lncRNA expression, mainly downregulated in Marsh 3C patients with respect to HCs. Among the 87 investigated transcripts, we focused on NEAT1 and TUG1.

Nuclear enriched abundant transcript 1 (NEAT1) is transcribed from a genetic locus called familial tumor syndrome multiple endocrine neoplasia (MEN) type I on human chromosome 11. There are two isoforms, 3.7-kb NEAT1_1 and 23-kb NEAT1_2, that differ in the 3′ end [19]. Both isoforms of NEAT1 are essential components of paraspeckles, nuclear bodies formed by RNAs and several proteins (more than 60) that are able to regulate gene expression by sequestering transcription factors [19].

Taurine-upregulated gene 1 (TUG1) is transcribed from a gene localized on chromosome 22 (22q12.2) and it is able to regulate gene expression through different mechanisms, since it interacts with Polycomb repressor complex and functions in the epigenetic regulation of transcription, but can also function in the cytosol sponging microRNAs [37]. Interestingly, this latter mechanism has been reported for microRNAs implicated in IL-1β, IL-6, IL-10 and TNFα signaling [21,22], and thus in processes involved in CD pathogenesis. The downregulation of NEAT1 and TUG1 initially seen with the array was confirmed with RT-qPCR on samples from both adult and pediatric patients in the active stage of CD with respect to their matched HCs. This downregulation, however, can be attributable to the destruction of the epithelium observed in Marsh 3C since, as demonstrated by laser microdissection experiments, both lncRNAs are mostly expressed in enterocytes.

On the contrary, an upregulation in NEAT1 and TUG1 expression was observed in ex vivo stimulation with PT-gliadin in the biopsies obtained from GFD patients but not in those from healthy subjects, thus showing that both these lncRNAs respond to gluten only in CD mucosa. The exposure of CD biopsies to PT-gliadin is able to trigger the activation of the immune response; this could involve innate immunity players mainly in the epithelium and the adaptive immunity ones in the lamina propria [2]. In fact, the analysis of the expression of three cytokines known to respond to gluten in CD pathogenesis, namely, IL-8, IL-15 and IFNγ [1], revealed an increase after PT-gliadin stimulation. Pearson analysis detected a significant correlation between NEAT1 and IL-15, as well as TUG1 and IL-15; ex vivo stimulation of HC biopsies confirmed an induction of NEAT1 expression by IL-15, but not by IL-8 or IFNγ, and no significant increment of TUG1. Thus, the activation of innate immunity is involved in NEAT1 expression regulation, but this involves IL-15 rather than IL-8. Interestingly, this finding is in agreement with data previously reported by Imamura et al. [20] that demonstrated the ability of NEAT1 to sequester into paraspeckles the splicing factor proline/glutamine-rich (SFPQ), a repressor of IL8 transcription, thus placing IL-8 downstream of NEAT1. To better characterize the interaction between IL-15 and NEAT1, we performed an in silico analysis that suggested the involvement of STAT3, a transcription factor common both to IL-15 (canonical) and IL-8 (non-canonical) signaling [24,25]. To better dissect a potential axis between cytokines and these lncRNAs, we employed an in vitro cell model. IL-15 treatment of HIEC-6 induced an increase in NEAT1 expression, STAT3 translocation into the nucleus and binding to a STAT3 binding region in the NEAT1 promoter; taken together, these data confirm the presence of an IL-15/STAT3/NEAT1 axis.

IL-15 is the trigger of innate immune response in the intestinal epithelium in CD, but the findings reported here shed some new light on the complexity of the cascade of regulatory mechanisms originating from gluten exposure. The upregulation of NEAT1 could represent a mechanism aiming to potentiate the immune response, for example, through the mechanism regulating IL-8 [20]; interestingly, TUG1 has also been implicated in the regulation of the inflammatory response, as demonstrated in an animal model of spinal cord injury, where TUG1 knockout prevented the activation of the TLR4/NF-κB/IL-1β pathway [38]. However, it must be remembered that TUG1 mainly acts through microRNA sponging, and Han et al. recently reported that TUG1 can regulate the TNF-induced cytokine production through the modulation of miR142-5p [21]. Differently from what was reported in the spinal cord injury model, in ulcerative colitis and HT29 cells, a decreased TUG1 expression was associated with increased cytokine production, and an overexpression of this lncRNA lead to increased cell survival.

Indeed, increased NEAT1 expression could also represent a defense mechanism triggered by gluten exposure and IL-15 production. In fact, the modulation of the expression of NEAT1 or TUG1 has been reported by various groups as part of a protective response to different stimuli [19,37]. NEAT1 increases after the induction of oxidative stress in HUVEC cells, and this process in mediated by P53 activation [39]. On the contrary, in the case of oxidative stress, increased survival has been associated with TUG1 downregulation, as observed in cardiomyocytes and neurons [40,41].

Last, but not least, both NEAT1 and TUG1 have been implicated in the regulation of the Wnt pathway, which represents a pivotal mechanism in the control of cell proliferation and differentiation in the intestine. A paper by Chen et al. dissects the role of NEAT1 in the regulation of β-catenin in glioblastoma [42], showing that the lncRNA was critical for increasing β-catenin nuclear transport, due to its ability to downregulate ICAT, GSK3B and Axin2. It must be noted that, even in this setting, the transcription of NEAT1 was regulated by STAT3, although in this case, this transcription factor was downstream of the EGF receptor. A similar effect of NEAT1 on β-catenin was also observed in non-small cell lung cancer cell lines, where NEAT1 KO by siRNA was able to reduce cell proliferation and invasion, as well as β-catenin transmigration in the nucleus [43]. Even TUG1 has a positive effect on the Wnt pathway, as demonstrated in colorectal cancer cells by Xiao et al. [44], where the reduction of TUG1 expression mediated by short hairpin was able to reduce β-catenin translocation into the nucleus, as well as cellular proliferation in vitro and in a xenograft model. The interaction between these two lncRNAs and the Wnt pathway surely deserves further study, since an activation of the Wnt/β-catenin axis has also been detected at the transcriptomic level in the duodenal mucosa of CD patients [45]. Although this could represent a response to gluten challenge, the correct regulation of this mechanism remains necessary in order to prevent uncontrolled proliferation.

In summary, this paper shows that NEAT1 and TUG1 expression is increased after gluten exposure only in celiac disease patients and that, for NEAT1, this process is induced by the production of IL-15 and STAT3 activation. The characterization of the downstream processes as well as the possible interaction with other molecules (such as microRNAs) could provide a better understanding of the molecular processes that are taking place in CD mucosa, possibly identifying patient subgroups and/or GFD response biomarkers.

## 4. Materials and Methods

### 4.1. Patients

Adult celiac patients and healthy controls were enrolled at IRCCS Cà Granda, Policlinico of Milano, whereas pediatric patients and matched controls were followed at San Gerardo’s Hospital of Monza. In both cases, written informed consent was obtained and the study was approved by the Ethical Committees of the respective institutions, in accordance with the Declaration of Helsinki. Only patients classified as Marsh 3 were regarded as celiac patients (CD); for stimulation experiments, patients on a gluten-free diet (GFD) for at least 12 months were included only if classified as Marsh 0.

### 4.2. Biopsy Processing and Ex Vivo Stimulation

For newly diagnosed CD patients and HCs, biopsies were snap frozen in liquid nitrogen and preserved at −80 °C until further processing. Specimens used for laser capture microdissection (LCM) were frozen in OCT.

For ex vivo experiments, biopsies were treated as previously described [46]; briefly, duodenal biopsies were rinsed three times with complete medium, and then incubated at 37 °C in a 5% CO_2_ atmosphere for four hours. Biopsies were incubated with medium alone or with medium supplemented with PT-gliadin, which was obtained from gliadin powder (Sigma-Aldrich, St Louis, MO, USA) digested with pepsin and trypsin enzymes, as already described [47]. For experiments involving cytokine stimulations, human recombinant IL-15, IL-8 and IFNγ (PeproTech^®^ EC, London, UK) were added to the medium. At the end of the incubation, biopsies were snap frozen and preserved at −80 °C.

### 4.3. Laser Capture Microdissection

HC biopsies were cut with a cryostat into 10 µm sections and quickly stained with hematoxylin and epithelium/non-epithelium sections were separated by laser cutting using an MMI UVcut^®^ (MMI, Eching, Germany). Each slide was cut for no longer than 30 minutes to prevent RNA degradation and the cut material was immediately frozen.

### 4.4. Cell Culture

HIEC-6 cells were obtained from Prof. Jean François Beaulieu of the University of Sherbrooke, Canada. They were cultured in OptiMem™ (Gibco™, Thermo Fisher Scientific, Whaltham, MA, USA) supplemented with fetal bovine serum, L-glutamine (EuroClone^®^, Pero MI, Italy), Hepes and hrEGF (Gibco™, Thermo Fisher Scientific, Whaltham, MA, USA) and seeded in 6-well plates at a cell density of 1.5 × 10^6^ cells/well. After five days, cells were stimulated for 24 h with hrIL-15 at different concentrations (1–10 ng/mL) or hrIL-8 (1–5 ng/mL) and then harvested either for RNA or protein extraction. Each experiment was performed in duplicate and repeated at least three times.

### 4.5. RNA Extraction and Gene Expression

Total RNA was extracted using the Direct-Zol RNA Miniprep Kit (Zymo Research, Irvine, CA, USA) after lysis with Trizol^®^ (Invitrogen™, Thermo Fisher Scientific, Waltham, MA, USA) following the manufacturer’s instructions. The lncRNA array was carried out with a Bio-Rad lncRNA Package, after a step of pre-amplification with an RT-PreAmp Kit (Bio-Rad, Hercules, CA, USA). In particular, to 96-well plates with pre-spotted primer couples for the transcripts of interest, 20 µL master mix and pre-amplified cDNA were added and the relative expression was normalized on GAPDH and HPRT. Gene expression analysis was performed on cDNA obtained with the High-Capacity cDNA Reverse Transcription Kit (Applied Biosystems™, Thermo Fisher Scientific, Waltham, MA, USA) and RT-qPCR was carried out with Luna^®^ Universal qPCR MasterMix (NEB, Ipswich, MA, USA) on a QuantStudio™7 Flex Real-Time PCR System (Applied BioSystem™, Thermo Fisher Scientific, Waltham, MA, USA).

The employed primers were the following:

HPRTF: TGAAAAGGACCCCACGAAGT, R: TTGAACTCTCATCTTAGGCTT;NEAT1F:CTTCCTCCCTTTAACTTATCCATTCAC, R: CTCTTCCTCCACCATTACCAACAATAC;TUG1F:ACGACTGAGCAAGCACTACC, R: CTCAGCAATCAGGAGGCACA;IL-8F: GGAAGGAACCATCTCACTGT, R: CCACTCTCAATCACTCTCAG;IL-15F: TTCACTTGAGTCCGGAGATGC; R: CCTCCAGTTCCTCACATTCTTTG;IFNγF: AAGAGTGTGGAGACCATCAAGG; R: ACTCCTTTTTCGCTTCCCTGT.

The housekeeping gene for sample normalization was HPRT and the 2^-ΔΔCt^ method with an external reference was used to determine the fold change in mRNA level. Each sample was analyzed in triplicate.

### 4.6. Protein Extraction and Western Blot

Cytosolic and nuclear proteins were extracted from HIEC-6 cells using the NE-PER™ Kit (Thermo Fisher Scientific™, Waltham, MA, USA) following the manufacturer’s instructions, and quantified with a Pierce™ Microplate BCA Protein Assay Kit (Thermo Fisher Scientific™, Waltham, MA, USA). Proteins were run on 10% bis-acrylamide gel in SDS buffer and transferred to a nitrocellulose membrane with 20% methanol buffer. The membrane was blocked with TTBS 0.1% + milk 5% and incubated overnight with the following primary antibodies: STAT3 (dilution 1:2000 #4904s, Cell Signaling Technology, Inc., Danvers, MA, USA), ACTB (dilution 1:1500 #A2066, Sigma Aldrich), followed by incubation with anti-rabbit HRP-conjugated antibody (dilution 1:10000 #7074, Cell Signaling Technology, Inc., Danvers, MA, USA). Blot density was quantified with ImageJ™ software (NIH, Bethesda, MD, USA).

### 4.7. Promoter Region Analysis

The promoter region of 3000 bp upstream of the transcription start site of NEAT1 and TUG1 were considered in the analysis. The raw FASTA sequence was obtained with Ensembl (NEAT1: ENSG00000245532; TUG1: ENSG00000253352) and directly analyzed with MatInspector and PROMO, looking for potential binding sites within a dissimilarity margin less than or equal to 15% [48,49,50]. Jaspar prediction software was employed to further identify STAT binding motifs on the NEAT1 promoter region, with a minimum relative profile score threshold of 80% [51]. Among the potential binding motifs of STAT3 on the NEAT1 promoter, there was the one previously reported by Cai et al. [52] that served as a basis to design DNA retardation assay probes (Figure S2).

### 4.8. DNA Retardation Assay

Putative STAT3 binding sites on the NEAT1 promoter region were predicted with Jaspar software, as already described, and are illustrated in Figure S2. Oligos were designed in this region as follows:

Biotinylated probe:

F:GGTGTTAACCAGGGAGAGGTTCCTGGCAGGAGTTCCTGTCAGATGCCATTTTCCATTCTG;
R:CAGAATGGAAAATGGCATCTGACAGGAACTCCTGCCAGGAACCTCTCCCTGGTTAACACC;

Cold probe:

F: AGGGAGAGGTTCCTGGCAGG;
R: CCTGCCAGGAACCTCTCCCT.

Reactions were set up with a LightShift™ Chemiluminescent EMSA Kit (Thermo Fisher Scientific™, Waltham, MA, USA) following the manufacturer’s instructions and samples were run on 5% non-denaturing acrylamide gels. Semi-dry transfer onto a nylon membrane was carried out with XCell Blot-Module (Invitrogen™, Thermo Fisher Scientific™, Waltham, MA, USA) followed by chemiluminescent detection.

### 4.9. Statistical Analysis

All statistical analyses were carried out with SYSTAT software (SPSS, Chicago, IL, USA). Evaluation of outliers was performed using the Grubbs and ROUT tests. An unpaired *t*-test was used to compare gene expression data from untreated biopsies, whereas a paired *t*-test was employed for ex vivo experiments. ANOVA multiple comparisons were used on data obtained from cells stimulated with more than one concentration of cytokines. Pearson correlation analyses were carried out to generate correlation data.

## Data Availability

Data available from the corresponding author upon reasonable request.

## Supplementary Materials

The following are available online at https://www.mdpi.com/1422-0067/22/3/1289/s1, Figure S1: IL-8 stimulations in HIEC-6 cells; Figure S2: Identification of STAT family binding motifs in NEAT1 promoter.
