# Supplementary material for: Gliadin, through the Activation of Innate Immunity, Triggers lncRNA NEAT1 Expression in Celiac Disease Duodenal Mucosa
Source: Int J Mol Sci. 2021 Jan 28;22(3):1289. doi: 10.3390/ijms22031289 (PMC7865487; doi:10.3390/ijms22031289)
Supplement: Supplementary file 1 [file ijms-22-01289-s001.pdf]

Supplementary Files

IL-8 stimulations in HIEC-6 cells

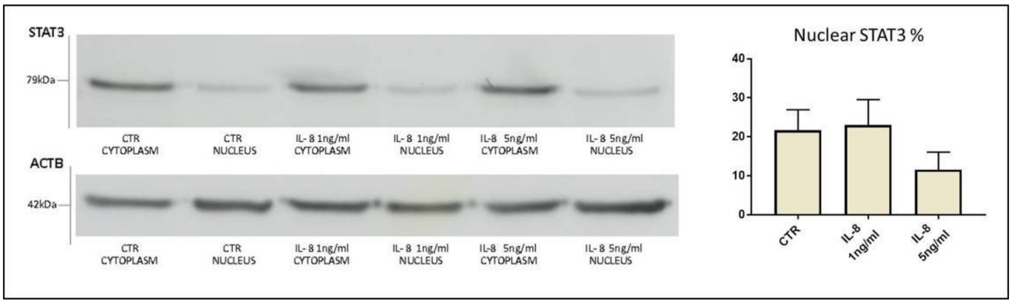

Figure S1. Translocation of STAT3 in the nucleus after IL-8 stimulation. Each sample was normalized on ACTB. Western blot image representative of three independent experiments.

Identification of STAT family binding motifs in NEAT1 promoter

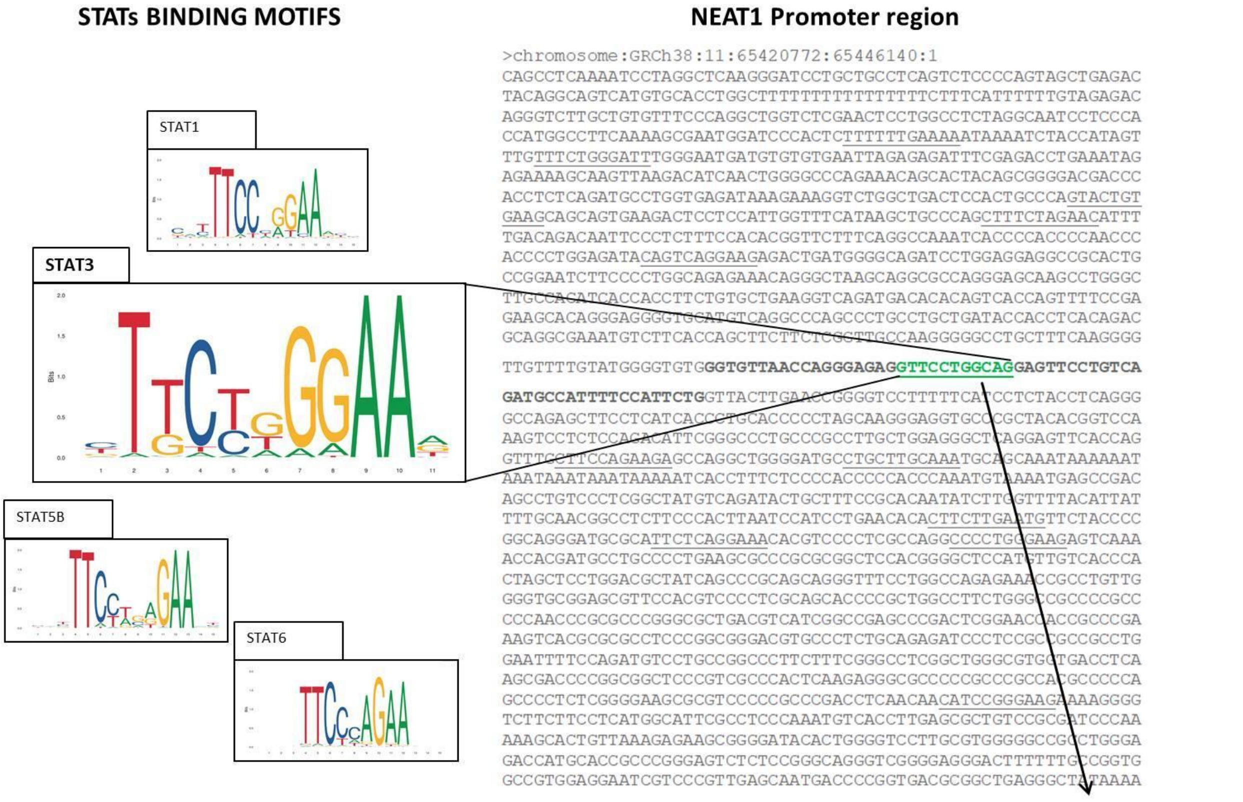

Matrix ID	Name	Score	Relative score	Sequence ID	Start	End	Strand	Predicted sequence
MA0144.2	STAT3	4.28612	0.849479192056	chromosome:GRCh38:11:65419774:65446140:	11778	1788	+	GTTCCTGGCAG

Figure S2. Identification of STAT family binding motif in NEAT1 promoter by Jaspar program. Common STATs binding motifs are reported on the left. On the right: raw FASTA sequence of NEAT1 promoter with STAT3 motifs underlined. The promoter region chosen to design gel shift biotinylated probes is in bold, the chosen STAT3 binding motif is highlighted in green. Predicted sequence location and relative score are reported as Jaspar prediction output.
